# Soluble Adenylyl Cyclase Is Required for Retinal Ganglion Cell and Photoreceptor Differentiation

**DOI:** 10.1167/iovs.16-19465

**Published:** 2016-09

**Authors:** Peter X. Shaw, Jiahua Fang, Alan Sang, Yan Wang, Michael S. Kapiloff, Jeffrey L. Goldberg

**Affiliations:** 1 Department of Ophthalmology, University of California San Diego, La Jolla, California, United States; 2 Department of Ophthalmology, First Hospital of Changsha, Changsha, Hunan Province, China; 3 Interdisciplinary Stem Cell Institute, Departments of Pediatrics and Medicine, Leonard M. Miller School of Medicine, University of Miami, Miami, Florida, United States; 4 Byers Eye Institute, Stanford University, Palo Alto, California, United States

**Keywords:** soluble adenylyl cyclase, retinal ganglion cell, retinal development, amacrine cell, photoreceptors

## Abstract

**Purpose:**

We have previously demonstrated that soluble adenylyl cyclase (sAC) is necessary for retinal ganglion cell (RGC) survival and axon growth. Here, we further investigate the role of sAC in neuronal differentiation during retinal development.

**Methods:**

*Chx10* or *Math5* promoter-driven Cre-Lox recombination were used to conditionally delete sAC from early and intermediate retinal progenitor cells during retinal development. We examined cell type–specific markers expressed by retinal cells to estimate their relative numbers and characterize retinal laminar morphology by immunofluorescence in adult and newborn mice.

**Results:**

Retinal ganglion cell and amacrine cell markers were significantly lower in the retinas of adult *Math5^cre^*/*sAC*^*fl*/*fl*^ and *Chx10^cre^*/*sAC*^*fl*/*fl*^ mice than in those of wild-type controls. The effect on RGC development was detectable as early as postnatal day 1 and deleting sAC in either Math5- or Chx10-expressing retinal progenitor cells also reduced nerve fiber layer thickness into adulthood. The thickness of the photoreceptor layer was slightly but statistically significantly decreased in both the newborn *Chx10^cre^*/*sAC*^*fl*/*fl*^ and *Math5^cre^*/*sAC*^*fl*/*fl*^ mice, but this reduction and abnormal morphology persisted in the adults in only the *Chx10^cre^*/*sAC*^*fl*/*fl*^ mice.

**Conclusions:**

sAC plays an important role in the early retinal development of RGCs as well as in the development of amacrine cells and to a lesser degree photoreceptors.

Understanding the signal transduction pathways controlling neuronal development and axon growth is an important step for developing therapies to treat neurodegenerative diseases. Cyclic adenosine monophosphate (cAMP) is a ubiquitous second messenger critical to survival and the axon growth of neurons.^1^ cAMP synthesis is catalyzed by a family of transmembrane (tmACs) and soluble (sAC) adenylyl cyclases.^2–4^ Unlike the nine tmACs that associate with G-protein coupled receptors and are activated by forskolin, sAC is sensitive to variations in intracellular concentrations of ATP, calcium, and bicarbonate.^5^ Activation by bicarbonate confers unique functions in CO_2_ and pH physiological sensing.^5^

Although sAC was originally detected in testis, later research confirmed that sAC is widely expressed in almost every mammalian tissue, where it is localized to the nucleus, mitochondria, and cytoplasm of cells.^6–8^ Immunofluorescence staining showed that sAC was abundant in most ocular tissues, including the cornea, the ciliary body, and throughout the layers of the neurosensory retina and the retinal pigment epithelium.^9^

In developing and adult retinal ganglion cells (RGCs), electrical activity promotes survival and axon growth by a cAMP-dependent mechanism^10,11^ that enhances RGC responsiveness to growth factors.^12^ In a subset of RGCs, blockade of tmACs decreased, but did not prevent calcium-dependent activation of the cAMP/PKA cascade.^13^ Our previous research showed that physiologic sAC activators, electrical activity, and bicarbonate significantly increased survival and axon growth of RGCs in vitro and that blocking sAC expression or activity decreased RGC survival in vitro and in vivo after optic nerve injury.^14^ Subsequent work suggested that the delivery of viral vectors designed to express sAC could promote RGC survival and regeneration after optic nerve injury.^15^

The function of sAC in retinal development remains largely unknown. In the present study, we aim to investigate whether sAC impacts the differentiation and development of retina and its neurons by eliminating sAC from all retinal progenitor cells very early in retinal development using *Chx10* promoter-driven Cre and *sAC* (*Adcy10*) flox/flox allele recombination. We have also conditionally removed sAC from retinal progenitor cells that have committed RGC fate and their progeny using *Math5* (*Atoh7*) promoter-driven Cre. We show the relevance of sAC to retinal development and differentiation using new sAC conditional knockout (cKO) mice by examining each retinal neuron cell type marker and the morphology of adult and newborn mouse retinas.

## Methods

### Animals

All animal procedures were performed in accordance with the ARVO Statement for the Use of Animals in Ophthalmic and Vision Research and were approved by Institutional Biosafety Committee and the Institutional Animal Care and Use Committee at the University of California, San Diego. Conditional gene deletion was achieved by crossing mice containing a conditional “floxed” sAC-C2 allele^16^ in which the second catalytic domain is flanked by loxP sites with *Chx10^cre^* trangenic (Jax mice stock no. 005105) or *Math5^cre^* knock-in mice (generous gift from Lin Gan).^17^ Genotyping of the sAC-C2 allele was by polymerase chain reaction as previously described.^7,16^ Age-matched litter mates without the *Chx10^cre^* or *Math5^cre^* allele served as controls. For counting and statistical analysis of adult or newborn postnatal day 1 (P1) mice retinas, we used 6 eyes from three mice of either sex in each experimental group.

### Western Blot

Adult and P1 mice were euthanized and retinas were dissected and lysed with lysis buffer (Cell Signaling Technology, Boston, MA, USA) containing 0.5 mM phenylmethanesulfonyl fluoride (Sigma-Aldrich Corp., St. Louis, MO, USA). Protein concentration was determined by bicinchoninic acid assay (Thermo Fisher Scientific, Grand Island, NY, USA). Samples (25 μg) were fractionated by sodium dodecyl sulfate- polyacrylamide gel electrophoresis in 4% to 20% gradient Tris (hydroxymethyl) aminomethane-glycine precast gels (Invitrogen, Life Technologies, Carlsbad, CA, USA) and transferred to a polyvinylidene difluoride membrane (Millipore, Billerica, MA, USA). The membrane was incubated for 1 hour in blocking solution containing 5% nonfat milk powder and 0.1% Tween-20, pH 7.6. This was followed by overnight incubation at 4°C in blocking solution containing rabbit primary antibodies against sAC (Abcam Ab82854, 1:50; Abcam, Cambridge, UK). Subsequently, the labeled proteins were visualized by incubation with a horseradish peroxidase–conjugated anti-goat or rabbit secondary antibody (1:2000; Santa Cruz Biotechnology, Dallas, TX, USA) followed by development with a chemiluminescence substrate for horseradish peroxidase (Thermo Fisher Scientific). The images of the Western blots were captured by GE imageQuant (GE Healthcare Biosciences, Pittsburgh, PA, USA). Relative band intensities were analyzed using ImageJ software http://imagej.nih.gov/ij/; provided in the public domain by the National Institutes of Health, Bethesda, MD, USA) and normalized to glyceraldehyde 3-phosphate dehydrogenase (GAPDH).

### Retinal Immunofluorescence Staining

Adult mouse tissues were fixed by cardiac perfusion under anesthesia using a saline rinse followed by 4% paraformaldehyde. Excised eyes of adult and newborn mice were fixed in 4% paraformaldehyde in 0.1 M phosphate-buffered saline (PBS) at 4°C overnight. A corneal puncture was performed to increase paraformaldehyde penetration into the eye.

Fixed retinas were incubated in 30% sucrose at 4°C overnight before mounting in an optimal cutting temperature compound (Sakura Finetek, Torrance, CA, USA) for frozen sectioning at 10 μm thickness. The sections were rinsed three times with PBS for 10 minutes each and then blocked in antibody buffer containing 0.2% Triton X-100 and 5% nonimmune serum derived from secondary antibody species for 60 minutes. Primary antibodies included rabbit polyclonal anti-ADCY10 antibody (ab203204; 1:50; Abcam), anti-Brn3a (AB5945), antirecoverin (AB5585) and antirhodopsin (MAB5356) monoclonal antibodies (EMD Millipore, Billerica, MA, USA), anti-β-tubulin monoclonal antibody (MMS-410P, Covance, San Diego, CA, USA), Pax6 (sc-7750) and PKC-α (sc-208) antibodies (Santa Cruz Biotech, Dallas, TX, USA), and anti-glutamine synthetase (G2781; Sigma Corporation). Primary antibodies were added in the above blocking buffer and incubated overnight at 4 °C, washed three times with PBS, and incubated with 4′,6-diamidino-2-phenylindole (DAPI), and Alexa fluorophore-conjugated secondary antibodies (1:500; Invitrogen) overnight at 4 °C. After three additional PBS washes, the sections were mounted and imaged by fluorescence microscopy.

### Retinal Neuron Quantification and Morphologic Analysis

Immunofluorescence-positive cells of the antibodies were quantified in each of the retinal layers relevant to that cell type. To minimize error from retinal eccentricity, central sections of retina including the optic disc were selected, and cells were counted at 100 μm from the center of the optic disc. A minimum of 18 images representing the six eyes from three mice were analyzed for each group, computing the average and standard deviation across the images and using the eye as a biological unit.

### Statistical Analysis

All data are expressed as mean ± standard deviation using the eye as a biological unit and averaging at least three sections' measurements per eye and performing Student's *t*-tests between the six cKO and six control eyes. For multiple-group comparisons, differences were evaluated using one-way analysis of variance followed by post hoc Dunnett's *t*-test. The statistical analysis was conducted in SPSS 20.0 software (IBM Corp., Armonk, NY, USA). Differences with a value of *P* < 0.05 were considered significant.

## Results

### Identification of sAC Knockout Mice

**Figure 1:**
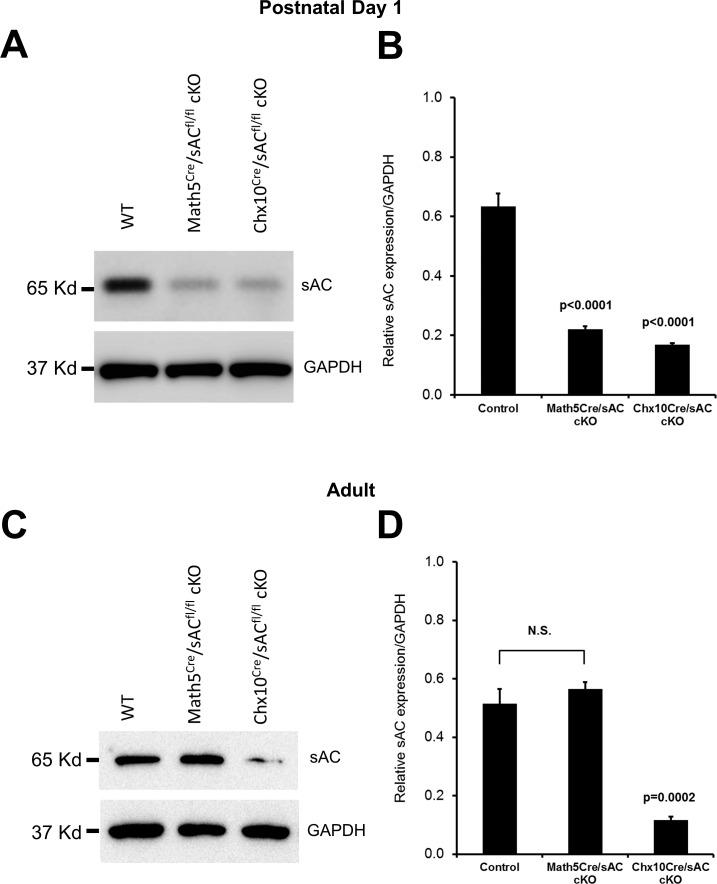
sAC protein expression is reduced in the retinas of sAC conditional knockout mice. Western blot analysis for sAC protein in control, *Math5^cre^*/*sAC*^*fl*/*fl*^, and *Chx10^cre^*/*sAC*^*fl*/*fl*^ mice retinas. (**A**, **C**) Representative Western blots and (**B**, **D**) relative expression levels of retinal sAC protein normalized to GAPDH from newborn (postnatal day 1, **A**, **B**) and adult (**C**, **D**) mice. *N* = 3; means ± standard deviations are shown. *P* values tested against control are indicated. n.s., nonsignificant.

We first confirmed the decrease in sAC protein after cre-lox-based recombination in early retinal development. *Chx10^cre^* and *Math5^cre^* alleles were selected to excise sAC from retinal progenitor cells in early or slightly later retinal development, respectively. Cell targeting for cre expression, particularly with *Chx10^cre^*, would be expected to excise sAC from most retinal progenitors, and their progeny of all retinal cell types, with the caveat that the Cre-mediated excision may not be 100% penetrant. In P1 mouse retinas, sAC protein expression was greatly decreased in both *Chx10^cre^*/*sAC*^*fl*/*fl*^ and *Math5^cre^*/*sAC*^*fl*/*fl*^ mice in comparison to wild-type littermates (Figs. 1A, 1B). In adults, the sAC protein expression in retinal tissues of *Chx10^cre^*/*sAC*^*fl*/*fl*^ mice was significantly decreased when compared with wild-type mice, whereas sAC was not decreased in *Math5^cre^*/*sAC*^*fl*/*fl*^ adult mice (Figs. 1C, 1D). These results demonstrate the significant reduction of the sAC protein in *Chx10^cre^* driven sAC conditional knockout in both newborn and adult retina. Residual sAC expression presumably derives from either Chx10-derived retinal cells in which recombination was incomplete or cell types not derived from retinal progenitors such as endothelial cells and nerve fiber layer astrocytes.^18^ In contrast, higher persistent sAC expression in adult *Math5^cre^* sAC cKO mice may be a result of a combination of incomplete cre expression penetrance, later onset of expression of Math5 compared to Chx10, and the fact that not all adult retinal neurons are ultimately of the Math5 lineage.^19^

### sAC Is Required for Amacrine Cell Differentiation, but Not for Bipolar Cells and Müller Cells

**Figure 2:**
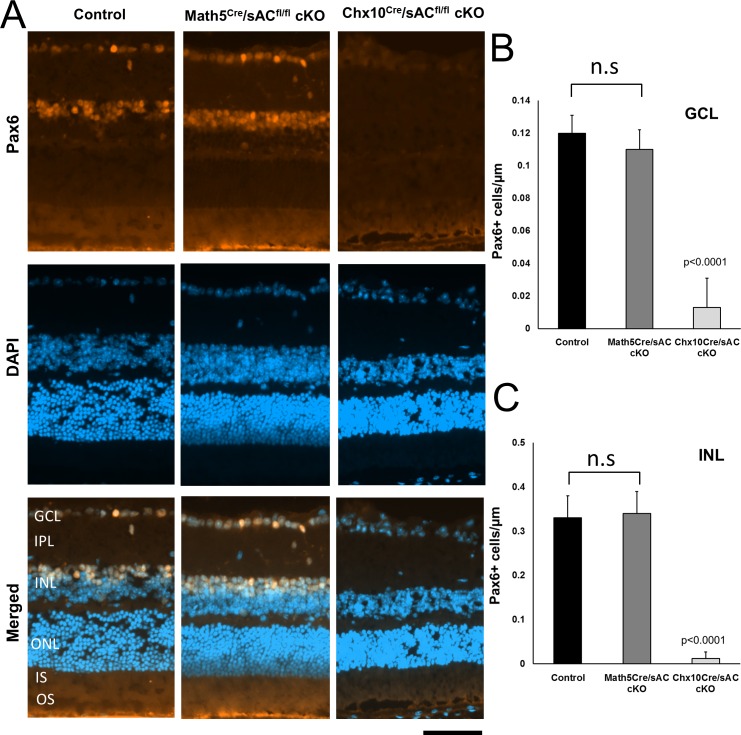
Pax6 expression is greatly reduced in the ganglion cell (GCL) and inner nuclear (INL) layers of *Chx10^cre^*/*sAC*^*fl*/*fl*^ cKO mice. (**A**) Immunofluorescence for Pax6 (*orange*) and DAPI for nuclei (*blue*) in retina cross-sections from wild-type controls (*left*), *Math5^cre^*/*sAC*^*fl*/*fl*^ (*middle*), and *Chx10^cre^*/*sAC*^*fl*/*fl*^ cKO mice (*right*). The retinal layers are indicated as follows: GCL, ganglion cell layer; IPL, inner plexiform layer; INL, inner nuclear layer; ONL, outer nuclear layer; IS/OS, inner segments/outer segments of photoreceptors. The Pax6+ cells in ganglion cell (**B**) and inner nuclear (**C**) layers were counted and expressed as mean ± standard deviation of the average of at least three sections per eye, 6 eyes (*n* = 6) per condition. *P* values are indicated on the top of the bar graphs. n.s., nonsignificant. *Scale bar*: 50 μm.

To investigate whether distinct retinal cell types were affected by the conditional deletion of sAC, we stained retinal cross-sections with monoclonal antibodies targeting specific markers of each retinal cell type expressed during development in combination with their histologic localizations. Although Pax6 is widely expressed in neuronal progenitors, in the adult retina Pax6 is only expressed in amacrine cells and RGCs. We identified Pax6+ cells in the inner nuclear layer (INL), representing amacrine cells, and in the ganglion cell layer (GCL), representing RGCs and displaced amacrine cells (Fig. 2A).

**Figure 3:**
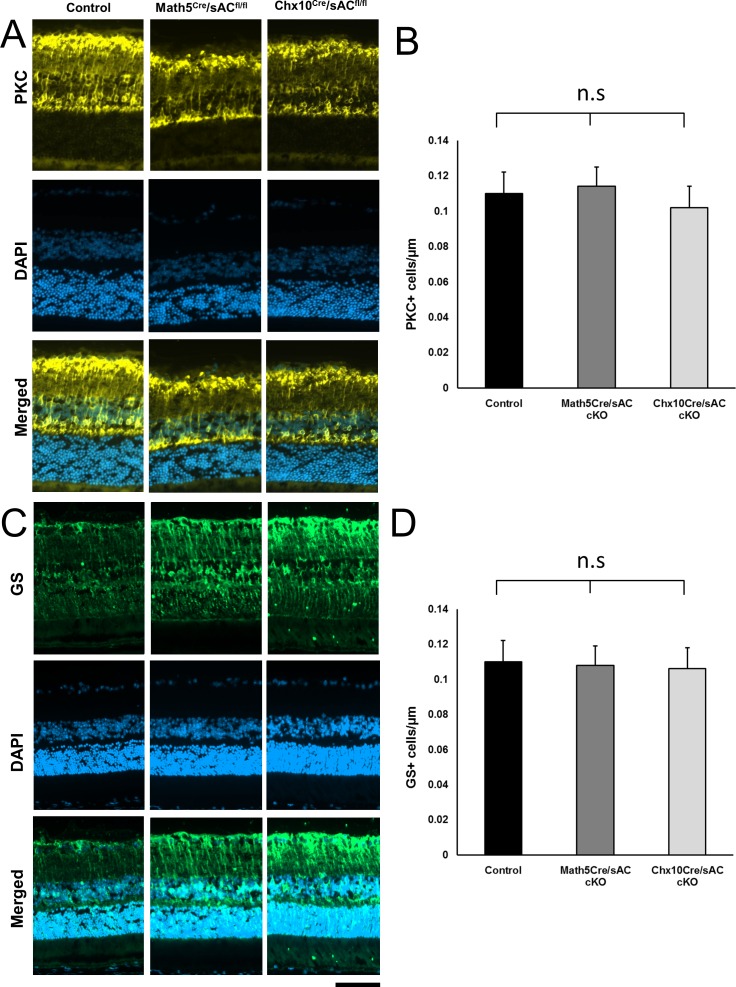
Conditional sAC knockout had no effect on retinal bipolar neurons or Müller glial cells. Immunofluorescence for PKC alpha (**A**, *yellow*) and GS (**C**, *green*) counterstained with DAPI for nuclei (*blue*) in retina cross-sections from wild-type controls (*left*), *Math5^cre^*/*sAC*^*fl*/*fl*^ (*middle*), and *Chx10^cre^*/*sAC*^*fl*/*fl*^ cKO mice (*right*). PKC+ (**B**) and GS+ cells (**D**) were counted and expressed as mean ± standard deviation (*n* = 6). n.s., nonsignificant. *Scale bar*: 50 μm.

We found that Pax6+ cells were significantly decreased in both the GCL and INL of *Chx10^cre^*/*sAC*^*fl*/*fl*^ mouse retinas in comparison with *Math5^cre^*/*sAC*^*fl*/*fl*^ mouse retinas. Thus, the conditional deletion of sAC by *Chx10^cre^* but not by *Math5^cre^* almost completely depleted Pax6+ cells from both the GCL and INL of retina (Figs. 2B, 2C). The almost complete depletion of Pax6+ cells in the INL indicates that sAC is necessary for amacrine cell differentiation. However, we did not observe changes in cell numbers or morphology of PKC-α+ bipolar cells or glutamine synthetase (GS+) Müller glial after conditional knockout of sAC by either *Math5* or *Chx10* promoter (Fig. 3), demonstrating cell type selectivity to the requirement for sAC in retinal differentiation.

### sAC Is Required for Photoreceptor Development

**Figure 4:**
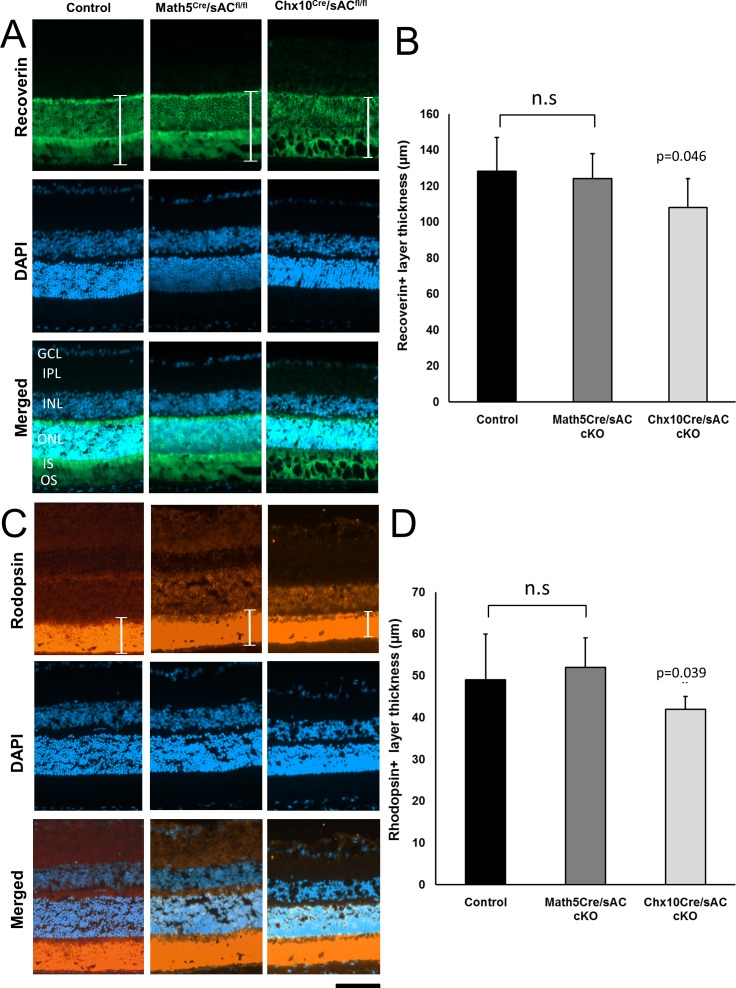
Conditional knockout of sAC reduces photoreceptor layer thickness. Immunofluorescence for recoverin (**A**, *green*) and rhodopsin (**C**, *orange*) counterstained with DAPI for nuclei (*blue*) in retina cross-sections from wild-type controls (*left*), *Math5^cre^*/*sAC*^*fl*/*fl*^ (*middle*), and *Chx10^cre^*/*sAC*^*fl*/*fl*^ cKO mice (*right*). The retinal layers are indicated as follows: GCL = ganglion cell layer; IPL = inner plexiform layer; INL = inner nuclear layer; ONL = outer nuclear layer; IS/OS = inner segments/outer segments of photoreceptors. The thickness of recoverin+ (**B**) and rhodopsin+ (**D**) layers (as marked with *brackets* in **A** and **C**) were measured and expressed as mean ± standard deviation. *P* values are indicated on the top of the bar graphs. n.s., nonsignificant. *Scale bar*: 50 μm.

To assess sAC's role in photoreceptor development, we stained adult retinas with cell type–specific markers in cKO and wild-type mice. Photoreceptor cell layers were stained with recoverin, a photoreceptor-specific calcium-binding protein.^20–22^ We detected a small but statistically significant decrease in the thickness of the photoreceptor layer in *Chx10^cre^*/*sAC*^*fl*/*fl*^ cKO mice, but not in *Math5^cre^*/*sAC*^*fl*/*fl*^ mice (Figs. 4A, 4B). Similarly, the rhodopsin+ layer reflecting the outer segments of rod photoreceptors was also decreased in the retina of *Chx10^cre^*/*sAC*^*fl*/*fl*^, but not in *Math5^cre^*/*sAC*^*fl*/*fl*^ mice (Figs. 4C, 4D). In addition, we observed a different morphology in the rhodopsin staining outside of the outer nuclear layer, indicating that the loss of sAC in retinal progenitor cells leads to morphologic changes in the photoreceptor layer in adult mice. These results showing the importance of sAC to photoreceptor development are consistent with observations that most adult photoreceptors are derived from Chx10+, but not Math5+, retinal progenitors.

### sAC Is Required for RGC Differentiation and Axon Growth

**Figure 5:**
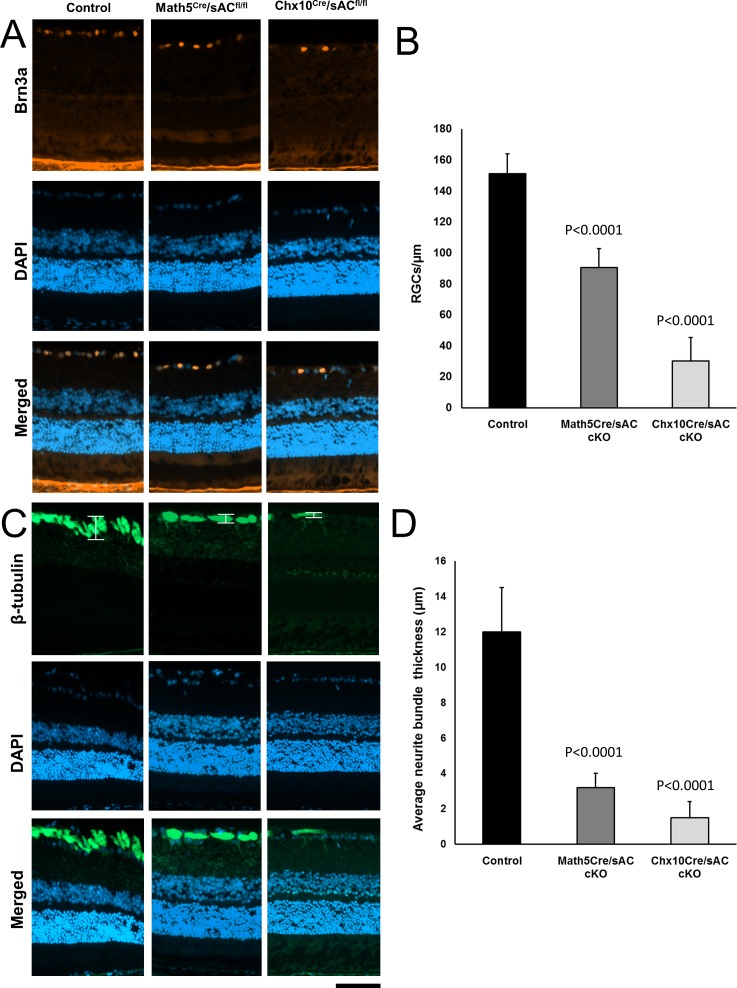
Retina ganglion cell numbers and optic nerve bundle thickness were reduced in the adult retina following *Math5^cre^* or *Chx10^cre^* sAC cKO. Immunofluorescence for Brn3a (**A**, *orange*) and β-tubulin (**C**, *green*) counterstained with DAPI for nuclei (*blue*) in retina cross-sections from wild-type controls (*left*), *Math5^cre^*/*sAC*^*fl*/*fl*^ (*middle*), and *Chx10^cre^*/*sAC*^*fl*/*fl*^ cKO mice (*right*). Brn3a+ cells (**B**) were counted, and the thickness of β-tubulin+ layers were measured (**D**) as marked with brackets in **C**. Data are expressed as mean ± standard deviation. *P* values are indicated on the top of the bar graphs. n.s., nonsignificant. *Scale bar*: 50 μm.

RGCs were identified by staining adult retinal cross-sections anti-Brn3a (AB5945), which stains nearly all RGCs. When compared with wild-type retinas, RGC numbers were significantly decreased in both *Math5^cre^*/*sAC*^*fl*/*fl*^ and *Chx10^cre^*/*sAC*^*fl*/*fl*^ mice, with a stronger effect evident for the *Chx10^cre^*/*sAC*^*fl*/*fl*^ cohort (Figs. 5A, 5B). Subsequent staining with β-tubulin, a marker of RGC somas and axons, was used to measure the thickness of the retinal nerve fiber layer to assess for changes in RGC axons. The thickness of axon fiber bundles were greatly reduced in the *Math5^cre^*/*sAC*^*fl*/*fl*^ and *Chx10^cre^*/*sAC*^*fl*/*fl*^ mice when compared with their wild-type counterparts (Figs. 5C, 5D). These data indicate that sAC significantly contributes to the differentiation and maturation of RGCs and their axons.

### sAC Role in Early RGC and Photoreceptor Differentiation

**Figure 6:**
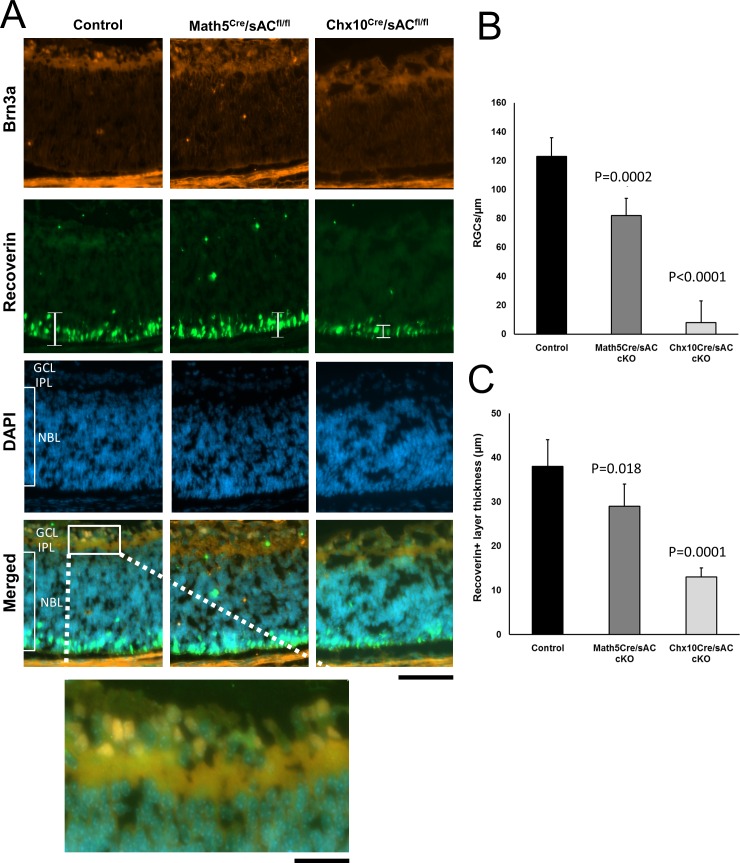
Retina ganglion cell numbers and photoreceptor layer thickness were reduced in the newborn mouse retina following *Math5^cre^* or *Chx10^cre^* sAC cKO. (**A**) Immunofluorescence for Brn3a (*orange*) and β-tubulin (*green*) counterstained with DAPI for nuclei (*blue*) in retina cross-sections from P1 wild-type controls (*left*), *Math5^cre^*/*sAC*^*fl*/*fl*^ (*middle*), and *Chx10^cre^*/*sAC*^*fl*/*fl*^ cKO (*right*) mice. (**B**) Brn3a+ cells (shown in the inset) were counted. The retinal layers of P1 mice are indicated as follows: GCL = ganglion cell layer; IPL = inner plexiform layer, and NBL = neuroblast layer. (**C**) Thickness of recoverin+ layers, as marked with brackets A, were measured. The data are expressed as mean ± standard deviation. *P* values are indicated on the top of the bar graphs. n.s., nonsignificant. *Scale bars*: 50 μm, 10 μm in inset.

To distinguish between sAC effects in early development and differentiation versus effects later in adulthood, including survival, we examined retinas from newborn (P1) mice stained with Brn3a and recoverin. Brn3a+ RGCs were significantly decreased in number for both *Math5^cre^* and *Chx10^cre^*-driven sAC cKO mice (Figs. 6A, 6B). The thickness of the recoverin+ photoreceptor layer was also significantly reduced in newborn sAC cKO mice (Figs. 6A, 6C). These results demonstrate that sAC plays an important role in early differentiation of RGC and photoreceptors.

## Discussion

Molecular signaling mechanisms that govern the differentiation and maturation of retinal neurons remain a subject of intense study.^23,24^ cAMP plays an important role in the differentiation of neural progenitor cells, for example, through cAMP response element binding protein phosphorylation.^25–27^ Forskolin, which elevates cAMP levels through transmembrane adenylate cyclases, also promotes the morphologic maturation of hippocampal neurons following differentiation from adult neural progenitor cells.^28^ Here we extend these observations from other central nervous system neurons by demonstrating the requirement for sAC in early retinal development and in the adult.

During eye development, retinal cells differentiate in a conserved sequence from a pool of multipotent progenitor cells directed by intrinsic properties and extrinsic cues.^29,30^ In the current study, conditional deletion of sAC generated by breeding sAC^fl/fl^ with *Chx10* promoter-driven Cre is expected to uniformly eliminate sAC from all or most retinal progenitor cells very early in retinal development, whereas *Math5* promoter-driven Cre is expected to delete sAC slightly later but still prior to RGC differentiation. The expression patterns of Chx10 and Math5 across retinal development have been previously studied in detail.^31–33^ Chx10, a POU (PIT-1, OCT1/2, unc-86) domain class 4 homeobox 2 transcription factor, is expressed earlier and more broadly in retinal progenitors,^31,34^ controlling the G1-phase cell cycle and essential for retinal progenitor cell proliferation^35,36^ and photoreceptor development.^37^ *Chx10* promoter activity drives expression in progenitor cells from early (E11) to later postnatal periods throughout the radial dimensions of the retina, although in the adult retina it is exclusively expressed in retinal bipolar cells.^38^ Math5 (also known as Atoh7) is a basic helix-loop-helix factor that regulates the cell cycle exit of multipotent retinal progenitors.^39^ *Math5* promoter activity in developing retina is detectable by E10 but peaks later, around E15 to E16,^17^ and is expressed in progenitor cells that also generate the progeny of nearly all retinal cell types,^39,40^ although Math5's expression is only required for RGC differentiation.^41^ Thus both lines contribute to the study of broad retinal deletion of sAC at early and later embryonic stages of retinal development.

The data presented here suggest that broad elimination of sAC in early retinal development impacts RGC development and also influences amacrine and photoreceptor differentiation as shown by differences in cell numbers and layer thickness, respectively. Although retinal sections were always analyzed at a fixed distance from the optic nerve to normalize for retinal thickness changes between the center and the periphery, it is possible that very small differences could be a result of slight differences in the angle of sectioning, although this would not be expected to explain large differences in RGC numbers. It is also possible that sAC deletion leads to a reduction of marker expression and cell size or outer segment length, but the correlation in such data leads us to favor a hypothesis that cell fate specification and differentiation are the loci of regulation. Because the deletion of sAC from retinal progenitor cells reduced RGC numbers with *Chx10*- more than *Math5*-driven Cre expression, we would hypothesize that earlier developmental expression of sAC is important for differentiation and that sAC activity is relevant in progenitor cells, as E10 to E11 deletion of sAC is occurring before the first RGCs differentiate from early progenitors. Furthermore, the detection of sAC effects on RGC number and photoreceptor layer thickness by P1 suggests that sAC plays a role in early differentiation and not just the survival of these cells into adulthood. This interpretation is also consistent with the loss of amacrine cells in the *Chx10*- but not the *Math5*-driven cKO. Amacrine cells are generated from retinal progenitors at the same time as RGCs and express a similar but not identical transcriptome.^42,43^ In contrast, the lack of sAC requirement for the differentiation of PKC-α+ bipolar cells and GS+ cells Müller glia demonstrates some selectivity that sAC is only required for the differentiation of certain cell types. Note that the broad deletion of sAC from retinal progenitors and their progeny does not allow one to identify whether specific progenitor subsets or cell–cell interactions are dependent on sAC for retinal cell type differentiation and remain an important question for future study.

Taken together, our results confirm that during early retinal development, sAC activity and expression in the retina play a critical role in retinal progenitor cell fate specification and RGC development. These data motivate further investigation to clarify the precise roles played by sAC during retinal development, in normal retinal function, and in therapeutic implications.
